# Supplementary material for: A Biomimetic Macrophage-Membrane-Fused Liposomal System Loaded with GVs-HV Recombinant Plasmid for Targeted Anti-Atherosclerosis Therapy
Source: Pharmaceutics. 2025 Dec 16;17(12):1618. doi: 10.3390/pharmaceutics17121618 (PMC12737165; doi:10.3390/pharmaceutics17121618)
Supplement: Supplementary file 1 [file pharmaceutics-17-01618-s001.zip › pharmaceutics-3969094-supplementary.pdf]

pharmaceutics

MDPI

Supplementary Materials: A Biomimetic Macrophage-Membrane-Fused Liposomal System Loaded with GVs-HV Recombinant Plasmid for Targeted Anti-Atherosclerosis Therapy

Yuelin Zhang, Wenting Gu, Kailing Yu, Qihong Chen, Hong Wang, Yinghui Wei, Hangsheng Zheng, Hongyue Zheng, Lin Liu and Fanzhu Li

Table S1. Different formulations of liposomes.

Lipids (mg)	DOTAP (%, *W/W*)											
	1%			3%			5%			10%		
Formula	1	2	3	4	5	6	7	8	9	10	11	12
PC S100	4	4	4	4	4	4	4	4	4	4	4	4
DSPE-PEG2000	1	2	1	1	2	1	1	2	1	1	2	1
Cholesterol	1	1	2	1	1	2	1	1	2	1	1	2

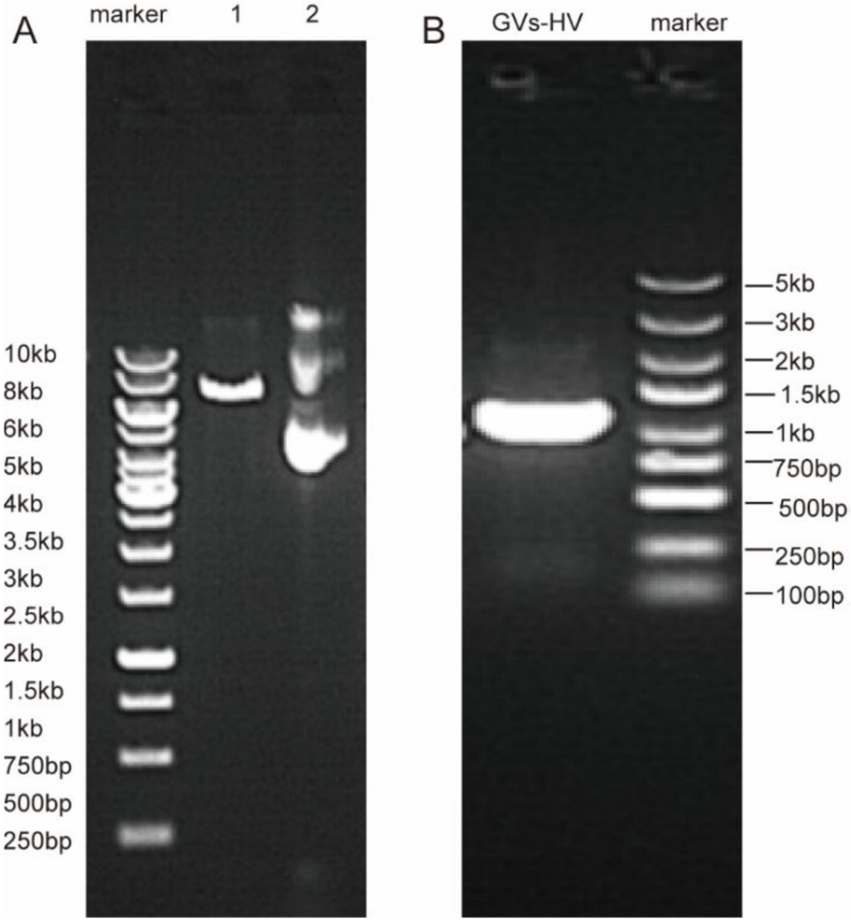

Figure S1. Construction and identification of GVs-HV recombinant fusion gene. (A) Results of agarose gel electrophoresis of GVs-HV recombinant plasmid fragment. Number 1 represents the

 https://doi.org/10.3390/pharmaceutics17121618

Enzyme-digested carrier, and number 2 represents the undigested vector. (B) Results of PCR of GVs-HV recombinant plasmid fragment. Different molecular weight bands of the marker have been marked in the figure.

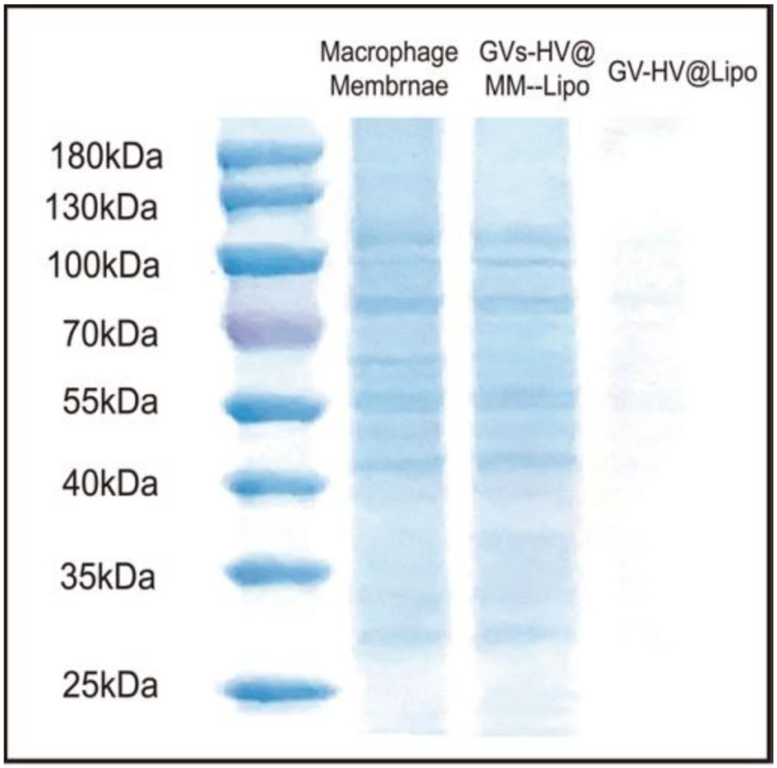

Figure S2. Coomassie brilliant blue results. Coomassie brilliant blue staining was used to investigate the ability of biomimetic hybrid vesicles to retain macrophage membrane proteins (Macrophage Membrane, GVs-HV@MM-Lipo, and GVs-HV@Lipo).

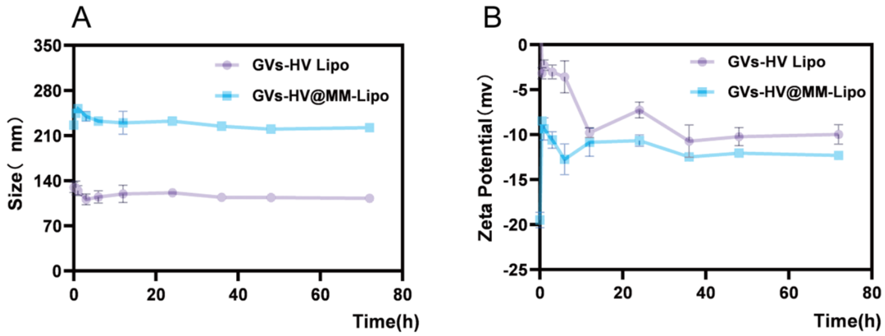

Figure S3. The particle size and Zeta Potential changes of GVs-HV@Lipo and GVs-HV@MM-Lipo in 10% serum during 72 h. (A) The particle size changes of GVs-HV@Lipo and GVs-HV@MM-Lipo in 10% serum (n = 3). (B) The Zeta Potential changes of GVs-HV@Lipo and GVs-HV@MM-Lipo in 10% serum (n = 3).

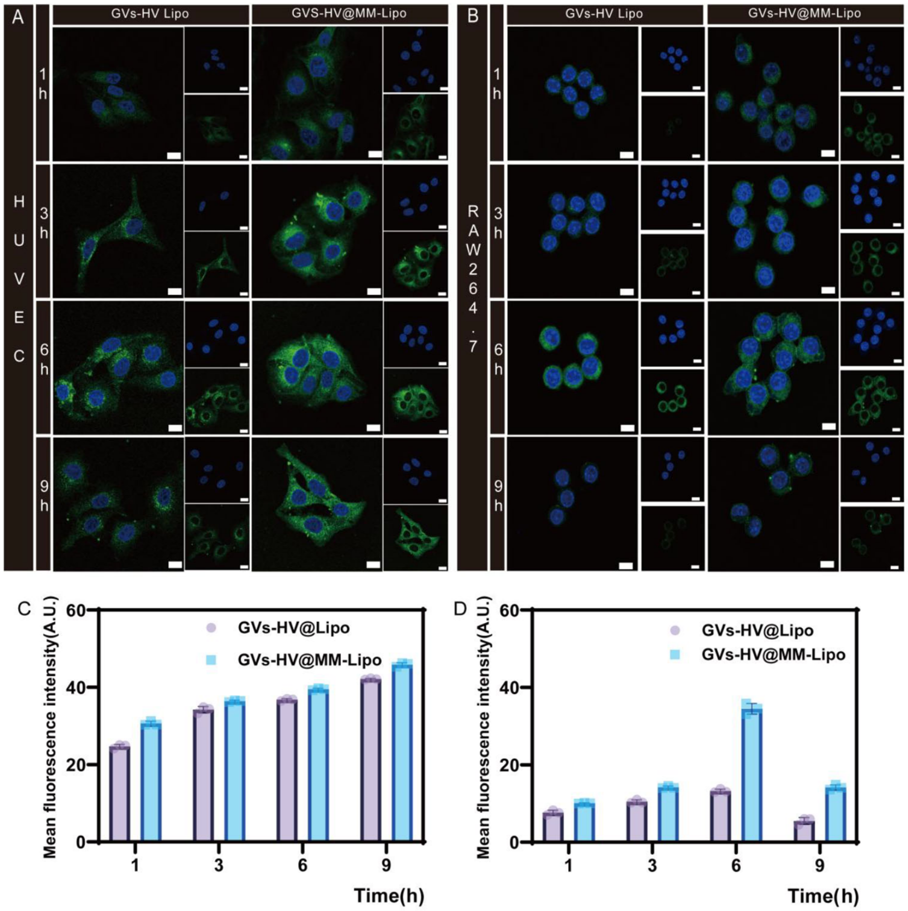

Figure S4. Time-dependent uptake and semi-quantitative fluorescence of GVs-HV @ MM-Lipo in HUVECs and Raw 264.7. (A) The time-dependent uptake of GVs-HV @ MM-Lipo in HUVECs (1,3,6,9 h) by confocal laser scanning. (B) The time-dependent uptake of GVs-HV @ MM-Lipo in Raw 264.7 (1,3,6,9 h) by confocal laser scanning. (C) The time-dependent uptake of GVs-HV @ MM-Lipo in HUVECs was semi-quantitatively quantified by fluorescence ($n = 3$). (D) The time-dependent uptake of GVs-HV @ MM-Lipo in Raw 264.7 was semi-quantitatively quantified by fluorescence ($n = 3$).

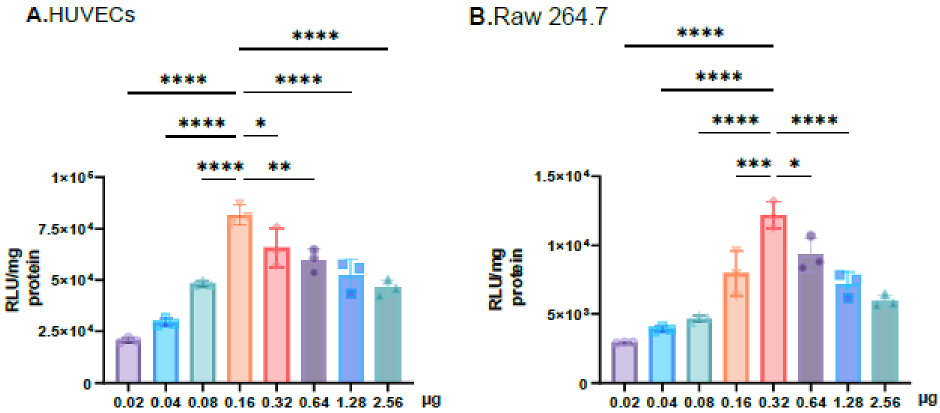

Figure S5. The transfection mass ratio of GVs-HV @ MM-Lipo was investigated. (A) The transfection mass ratio of GVs-HV @ MM-Lipo in HUVECs was investigated by firefly luciferase kit. (B) The transfection mass ratio of GVs-HV @ MM-Lipo in Raw 264.7 was investigated by firefly luciferase kit. Statistical analysis was performed by one-way ANOVA. *P ≤ 0.05, **P ≤ 0.01, and ***P ≤ 0.001.

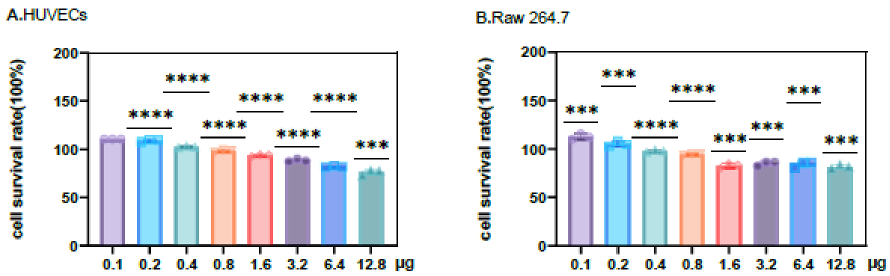

Figure S6. The cytotoxicity of GVs-HV @ MM-Lipo in HUVECs and Raw 264.7 was detected by MTT assay. (A) Cytotoxicity of GVs-HV @ MM-Lipo in HUVECs. (B) Cytotoxicity of GVs-HV @ MM-Lipo in Raw 264.7. Statistical analysis was performed by one-way ANOVA. ***P ≤ 0.001.

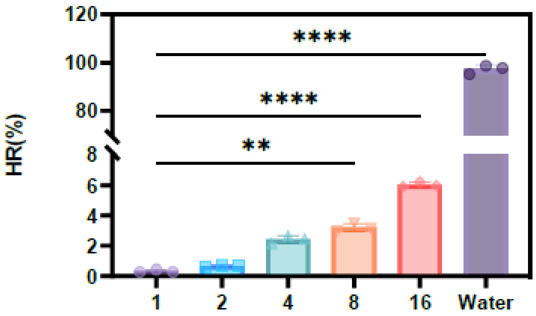

Figure S7. Hemolysis assay**.** In vitro safety of GVs-HV @ MM-Lipo. Statistical analysis was performed by one-way ANOVA. **P ≤ 0.01, and ***P ≤ 0.001.

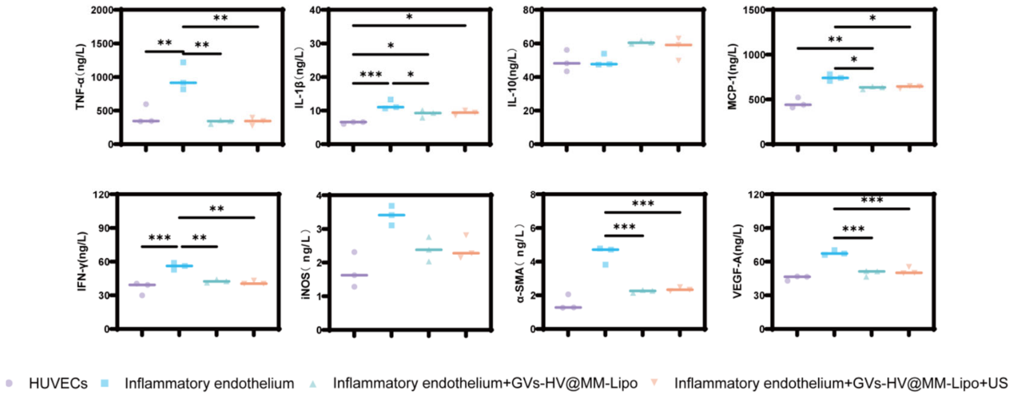

Figure S8. Differences in inflammatory factors and cytokines in the supernatant after co-culturing HUVEC cells with various experimental groups. The levels of inflammatory factors and chemokines (TNF-α, IL-1β, IL-10, MCP-1, IFN-γ, iNOS, α-SMA, VEGF-A) in HUVECs treated with GVs-HV @ MM-Lipo were detected. Statistical analysis was performed by one-way ANOVA. *P ≤ 0.05, **P ≤ 0.01, and ***P ≤ 0.001.

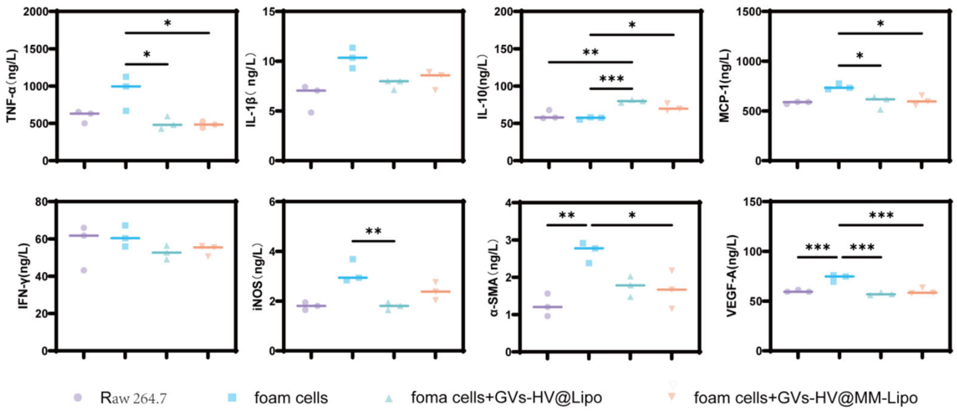

Figure S9. Differences in inflammatory factors and cytokines in the supernatant after co-culturing Raw 264.7 cells with various experimental groups. The levels of inflammatory factors and chemokines (TNF-α, IL-1β, IL-10, MCP-1, IFN-γ, iNOS, α-SMA, VEGF-A) in Raw 264.7 treated with GVs-HV @ MM-Lipo were detected. Statistical analysis was performed by one-way ANOVA. *P ≤ 0.05, **P ≤ 0.01, and ***P ≤ 0.001.

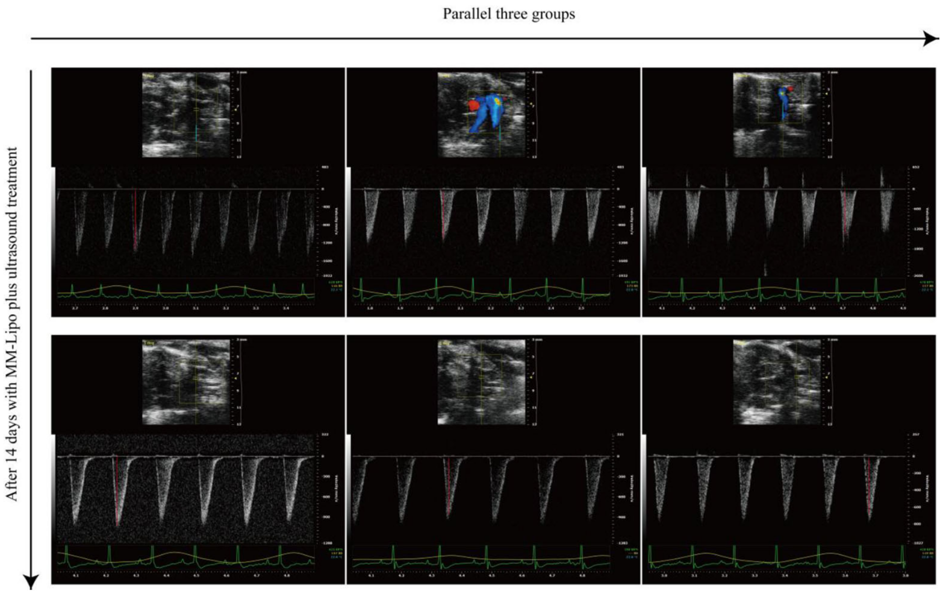

Figure S10. Ultrasound imaging of aortic arch blood flow. Ultrasound imaging of aortic arch blood flow in atherosclerotic mice before and after treatment with GVs-HV@MM-Lipo+US (n = 3).

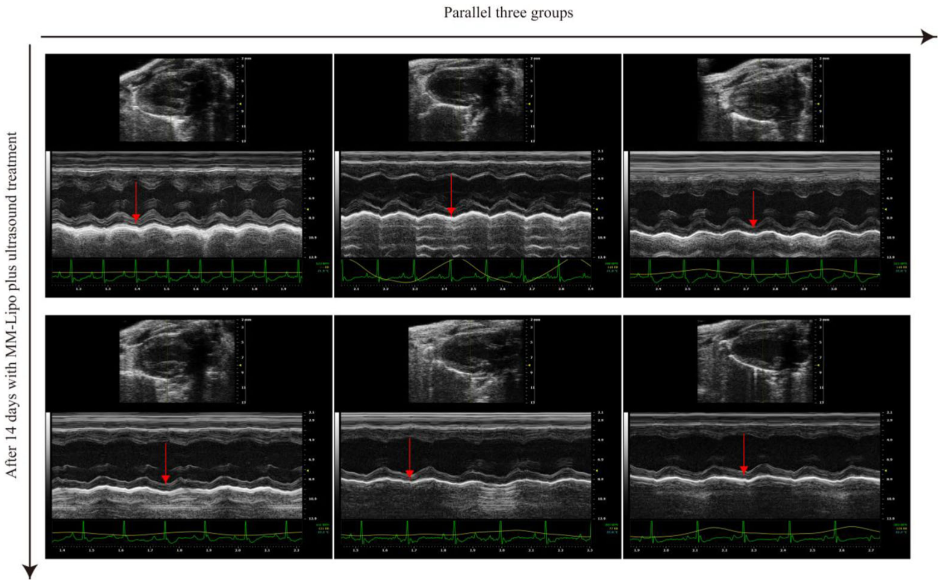

Figure S11. Ultrasonic imaging of ventricular wall thickness. Ultrasonic imaging of ventricular wall thickness in atherosclerotic mice before and after treatment with GVs-HV@MM-Lipo+US (n = 3).

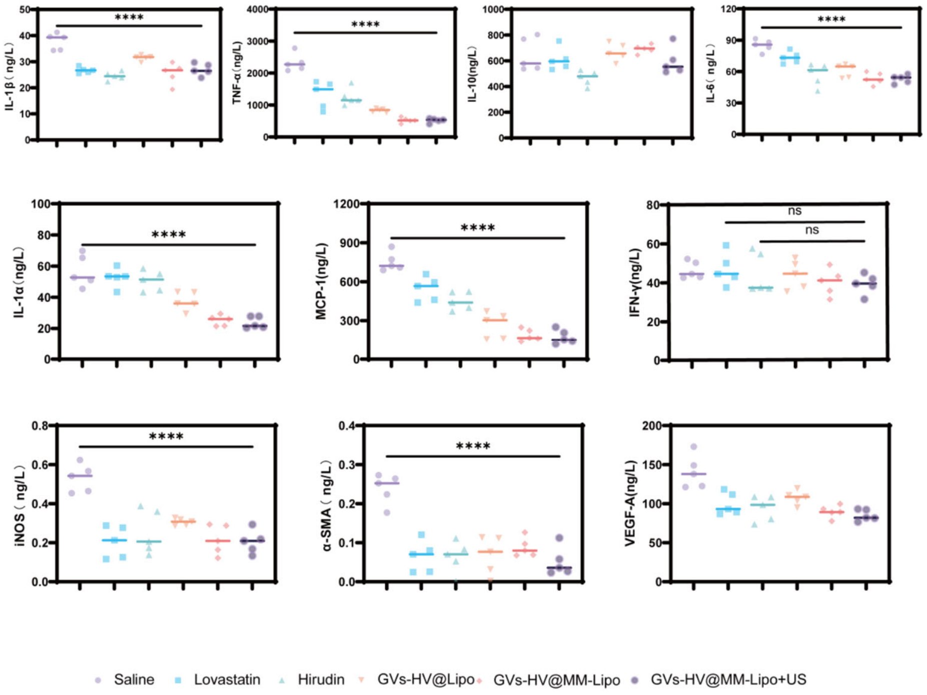

Figure S12. Differences in inflammatory factors and cytokines in atherosclerotic mice with various experimental groups. Detection of inflammatory factors and chemokines (IL-1β, TNF-α, IL-10, IL-6, IL-1α, MCP-1, IFN-γ, iNOS, α-SMA, VEGF-A) in atherosclerotic mice in different treatment groups (Saline, Lovastatin, Hirudin, GVs-HV@MM-Lipo, GVs-HV@MM-Lipo+US). Statistical analysis was performed by one-way ANOVA. ****P ≤ 0.0001.

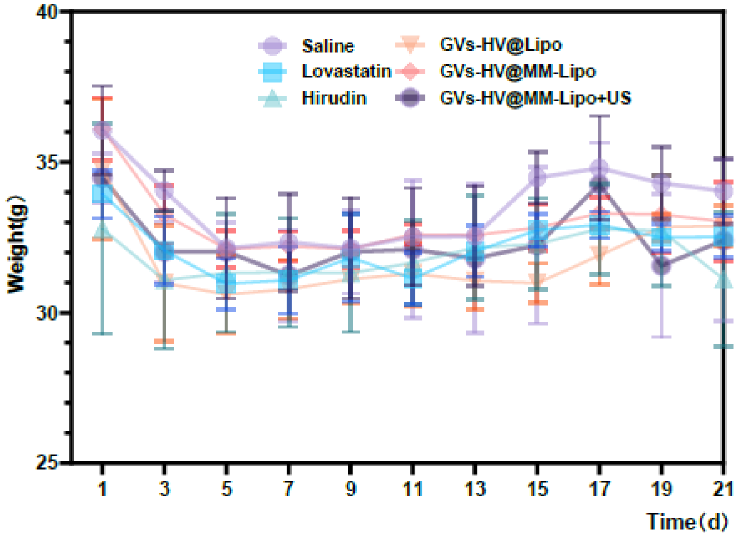

Figure S13. Body weight changes**.** Changes in body weight of atherosclerotic mice in different groups during treatment (Saline, Lovastatin, Hirudin, GVs-HV@Lipo, GVs-HV@MM-Lipo, GVs-HV@MM-Lipo+US).

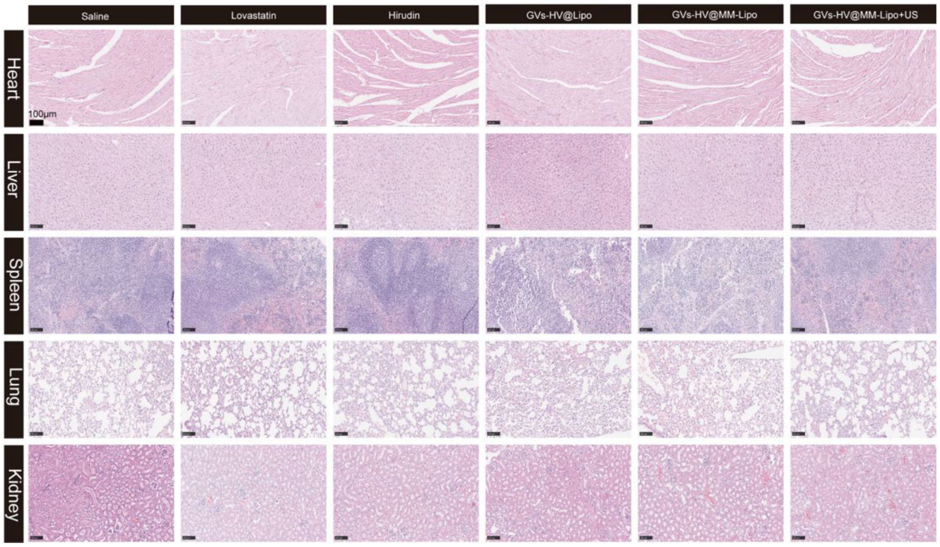

Figure S14. Safety assessment. Safety detection of heart, liver, spleen, lung and kidney in atherosclerotic mice in different treatment groups (Saline, Lovastatin, Hirudin, GVs-HV@Lipo, GVs-HV@MM-Lipo, GVs-HV@MM-Lipo+US). Scale Bar = 100 μm.

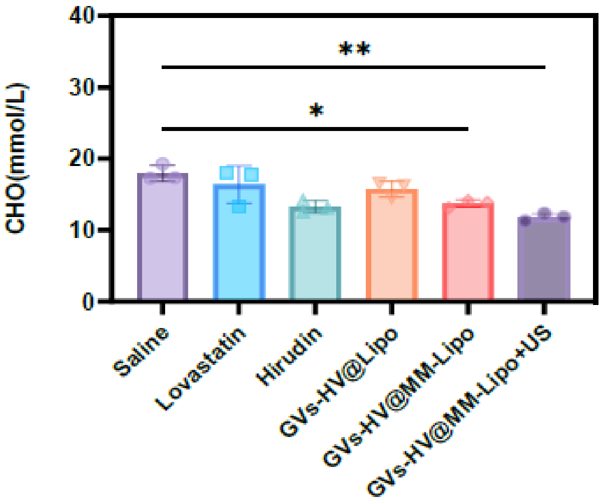

Figure S15. Changes of CHO levels. Changes of CHO levels in different groups of atherosclerotic mice during treatments (Saline, Lovastatin, Hirudin, GVs-HV@Lipo, GVs-HV@MM-Lipo, GVs-HV@MM-Lipo+US). Statistical analysis was performed by one-way ANOVA. $^{*}P \leq 0.05$, $^{**}P \leq 0.01$.

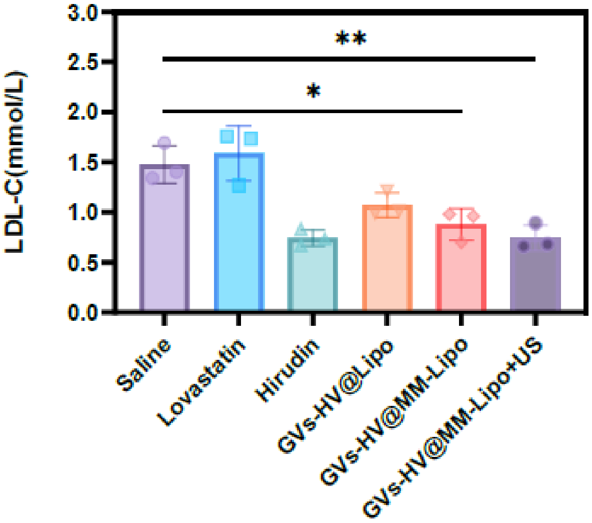

Figure S16. Changes of LDL-C levels. Changes of LDL-C levels in different groups of atherosclerotic mice during treatments (Saline, Lovastatin, Hirudin, GVs-HV@Lipo, GVs-HV@MM-Lipo, GVs-HV@MM-Lipo+US). Statistical analysis was performed by one-way ANOVA. $^{*}P \leq 0.05$, $^{**}P \leq 0.01$.

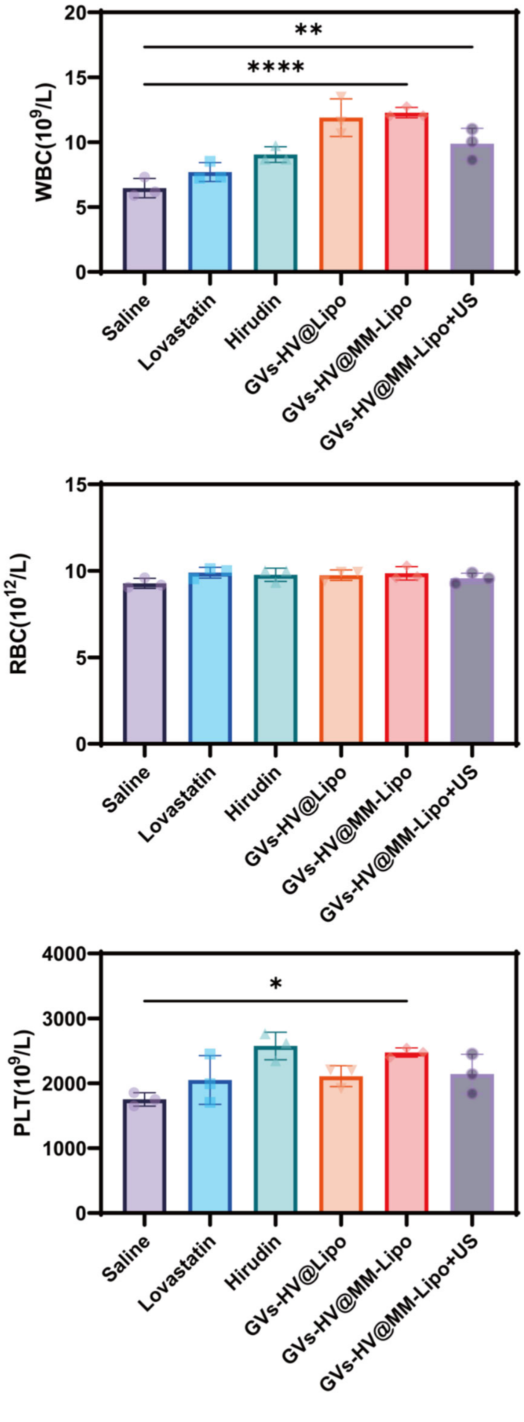

Figure S17. Blood routine test. Blood routine test of atherosclerotic mice in different treatment groups (Saline, Lovastatin, Hirudin, GVs-HV@Lipo, GVs-HV@MM-Lipo, GVs-HV@MM-Lipo+US). Statistical analysis was performed by one-way ANOVA. *$P \leq 0.05$, **$P \leq 0.01$, and ****$P \leq 0.0001$.
